# A systematic review and meta-analysis on impulse-control disorders in patients with pituitary adenomas exposed to dopamine agonists

**DOI:** 10.3389/fendo.2026.1869083

**Published:** 2026-08-06

**Authors:** Rodrigo Bilbao, José Serralunga, Nicolás Loschiavo, Renata Altuna, Joaquín Manfre, Francisco Capani, Matilde Otero-Losada, Osvaldo J. Ponzo, Santiago Perez-Lloret

**Affiliations:** 1 Departamento de Fisiología, Facultad de Medicina, Universidad de Buenos Aires (UBA), Buenos Aires, Argentina; 2 Centro de Altos Estudios en Ciencias Humanas y de la Salud, Universidad Abierta Interamericana, Consejo Nacional de Investigaciones Científicas y Técnicas, CACEIHS. UAI-CONICET, Buenos Aires, Argentina; 3 Instituto de Ciencias Biomédicas, Facultad de Ciencias de la Salud, Universidad Autónoma de Chile, Santiago, Chile; 4 Instituto Universitario de Ciencias de la Salud, Fundación H.A Barceló, Consejo Nacional de Investigaciones Científicas y Técnicas, Buenos Aires, Argentina

**Keywords:** dopamine agonists, hyperprolactinemia, impulse-control disorders, pharmacovigilance, pituitary adenomas

## Abstract

**Background:**

Patients exposed to dopamine agonists (DAs) may develop impulse-control disorders (ICDs), as shown in Parkinson’s Disease. Patients with pituitary adenomas treated with DAs may also have a higher frequency of ICDs.

**Objective:**

We compared the frequency of ICDs or impulsiveness in patients with pituitary adenomas treated with DAs versus patients with pituitary adenomas not exposed to DAs or healthy controls.

**Methods:**

Observational studies published in any language up to June 30^th^, 2026 were searched for in PubMed, EMBASE, EBSCO, LiLACS, SCOPUS, and WOS databases. We compared the occurrence of ICDs and impulsiveness scale scores between patients exposed to and not exposed to DAs.

**Results:**

Out of 14 studies identified, 10 reported the association between DAs and ICDs, and 6 reported the impact on the Barratt Impulsiveness Scale. DAs were significantly associated with ICDs (random-effects models, OR, 95% CI: 2.39, 1.78-3.21; I^2^ = 0%, n=1247; p<0.01) regardless of age, sex, cabergoline dose, or treatment duration. There were no indications of publication bias. Exposure to DAs was significantly associated with hypersexuality (3.11, 1.93-5.02, p<0.01, I^2^ = 0%) and punding (2.53, 1.34-4.78, p<0.01, I^2^ = 27.6%), but not with pathological gambling (1.94, 0.84-4.49, p=0.12, I^2^ = 0%) or compulsive shopping (1.46, 0.59-3.36, p=0.41, I^2^ = 61.3%).

**Conclusion:**

Patients with pituitary adenomas treated with DAs showed a higher frequency of ICDs. Studies to confirm these findings and investigate the risk factors for ICDs in this patient population are warranted.

## 1 Introduction

Prolactinomas are the most common type of functioning pituitary adenomas and the most common cause of non-physiological hyperprolactinemia, representing 40–60% of all pituitary adenomas, with an estimated prevalence of up to 100 cases per 100,000 people in the general population (1). Clinically, they are associated with galactorrhea, gonadal dysfunction, and tumoral mass effect, contributing to reduced quality of life and healthcare costs related to long-term endocrine care (2). The first-line therapy is oral administration of dopamine agonists (DAs), often cabergoline or bromocriptine (3). These act on dopamine D2 receptors — of the G-protein-coupled receptors family — and inhibit prolactin release via Gi protein activation in pituitary lactotrophs.

DAs have several adverse effects due to the widespread distribution of dopamine receptors, including nausea, vomiting, orthostatic hypotension, leg edema, headaches, daytime somnolence, and sleep disorders (4). Neuropsychiatric events have been increasingly recognized as well. Impulse control disorders (ICDs) are the most frequently described, characterized by compulsive/uncontrollable urges like pathological gambling, hypersexuality, compulsive shopping, punding, and others (5). These behaviors are thought to result from dopaminergic overstimulation of the mesolimbic reward system, particularly projections from the ventral tegmental area to the nucleus accumbens and may be more pronounced with D3 (e.g., ropinirole or pramipexole) than with D2 agonists.

ICDs observed in Parkinson’s Disease and other neurological conditions — typically found in patients taking high doses of DAs — have also been reported for lower doses recently (5). Dopamine dysregulation in the mesolimbic reward pathway, involving the overstimulation of D_2_ and D_3_ receptors, drives the loss of inhibitory control and heightened reward-seeking seen in ICDs (6). The rising number of reports linking DA exposure to ICDs in patients with pituitary adenomas indicates that individuals with endocrine disorders treated with DAs may also face an elevated risk of developing ICDs. This trend underscores the importance of conducting a systematic and comprehensive assessment of these potential adverse effects in this patient population. Because DAs are prescribed not only for prolactinomas but also for selected patients with other pituitary adenomas, and because most available studies report outcomes for DA-treated patients without distinguishing tumor subtype, this systematic review and meta-analysis aimed to compare the frequency of ICDs or impulsiveness in patients with pituitary adenomas of any subtype treated with DAs with that in patients not exposed to DAs or healthy controls.

## 2 Methods

### 2.1 Search strategy

This Systematic Review and Meta-analysis is reported according to the Guidelines of Preferred Reporting Items for Systematic reviews and Meta-Analyses (PRISMA) 2020 (7). The protocol for this study was registered at PROSPERO (CRD420250654677).

We searched the MEDLINE, EMBASE, EBSCO, LiLACS, SCOPUS, and WOS databases for studies published in any language through June 30^th^, 2026. The complete search strategy is shared online as **Supplementary Material** (**Supplementary Table E-1**). Reference lists of relevant reviews and original articles were also considered for more studies.

An initial screening of titles and abstracts was conducted collaboratively by all authors to identify potentially relevant studies. Records were evaluated according to predefined eligibility criteria.

### 2.2 Selection criteria

We defined PECOS as: (P) Patients with pituitary adenomas; (E) DAs at any dose and treatment duration; (C) other treatments or no treatment; (O) ICDs of any type; pathological gambling, compulsive shopping, hypersexuality, and punding were targeted specifically; Impulsivity as measured by any scale; (S) observational studies, including cross-sectional, case-control, or cohort studies.

We included observational studies of any kind that assessed the relationship between exposure to DAs and ICDs in patients with prolactin-producing or non-functioning pituitary adenomas. ICDs could be diagnosed using the DSM-4/5, the International Classification of Diseases (ICD) 10/11, any validated psychometric tool, or any other criteria. Studies using validated scales of impulsiveness or impulse-control deficits were also included. Full-text eligibility assessment was performed independently by pairs of reviewers (J.S., N.L., R.A., J.A.). Disagreements were resolved through consultation with a third reviewer (R.B.; S.P.L.). Cohen’s kappa ranged between 0.6 and 0.9, revealing a substantial agreement between examiners.

### 2.3 Data extraction and quality assessment

We retrieved the following information from each study: authors, year of publication, country, ICDs assessment tools, type, DAs dosage and treatment duration, study design, follow-up duration (if any), sample size, participants’ mean age and male proportion, risk factors for ICDs like psychiatric history or neurological disease, ICDs familial background, alcoholism, smoking, drug misuse, and obesity. We assessed the risk of bias using the ROBINS-I (Risk Of Bias In Non-randomized Studies of Interventions) scale (8).

Data extraction was performed by one reviewer and independently verified by a second reviewer. Any discrepancies were resolved through discussion among the authors.

### 2.4 Outcomes and summary measures

The primary outcome was the association between any ICD and exposure to DAs. The summary measure was the Odds ratio with a 95% confidence interval.

Secondary outcomes were the association of DAs with specific ICDs like pathological gambling, hypersexuality, and others, and impulsivity rating scales. For the latter, results were expressed as mean differences. Missing data was not imputed.

### 2.5 Statistical analysis

#### 2.5.1 Models

We used Mantel-Haenszel and inverse-variance estimators to calculate summary measures in fixed-effects models for binary and numeric variables, respectively. Random-effects models were calculated using the DerSimonian-Laird estimator.

#### 2.5.2 Heterogeneity assessment

Heterogeneity, was assessed by the Q-test and I^2^ statistic (9). Meta-regression was used to assess the impact samples’ mean age and male proportion, cabergoline dose, and cabergoline treatment duration.

#### 2.5.3 Subgroup and sensitivity analysis

Subgroup analysis was used to compare the strength of association when the control group comprised patients not exposed to DAs or healthy controls. If a single study provided data for both subgroups, each subgroup included half of the sample exposed to DAs.

A Leave-One-Out sensitivity analysis was performed to assess the impact of each study on the global effect. We also performed sensitivity analyses focusing on studies involving exclusively patients with prolactin-producing pituitary adenomas and studies that employed validated tools for the ICDs screening.

#### 2.5.4 Publication bias

Funnel plots were used to evaluate publication bias. The Harbord test was used to formally assess the presence of this bias.

#### 2.5.5 Software

All statistical analyses were performed using R version 4.1.6 (R Foundation for Statistical Computing, Vienna, Austria) with the packages tidyverse, meta, and metasens (10).

## 3 Results

**Figure 1:**
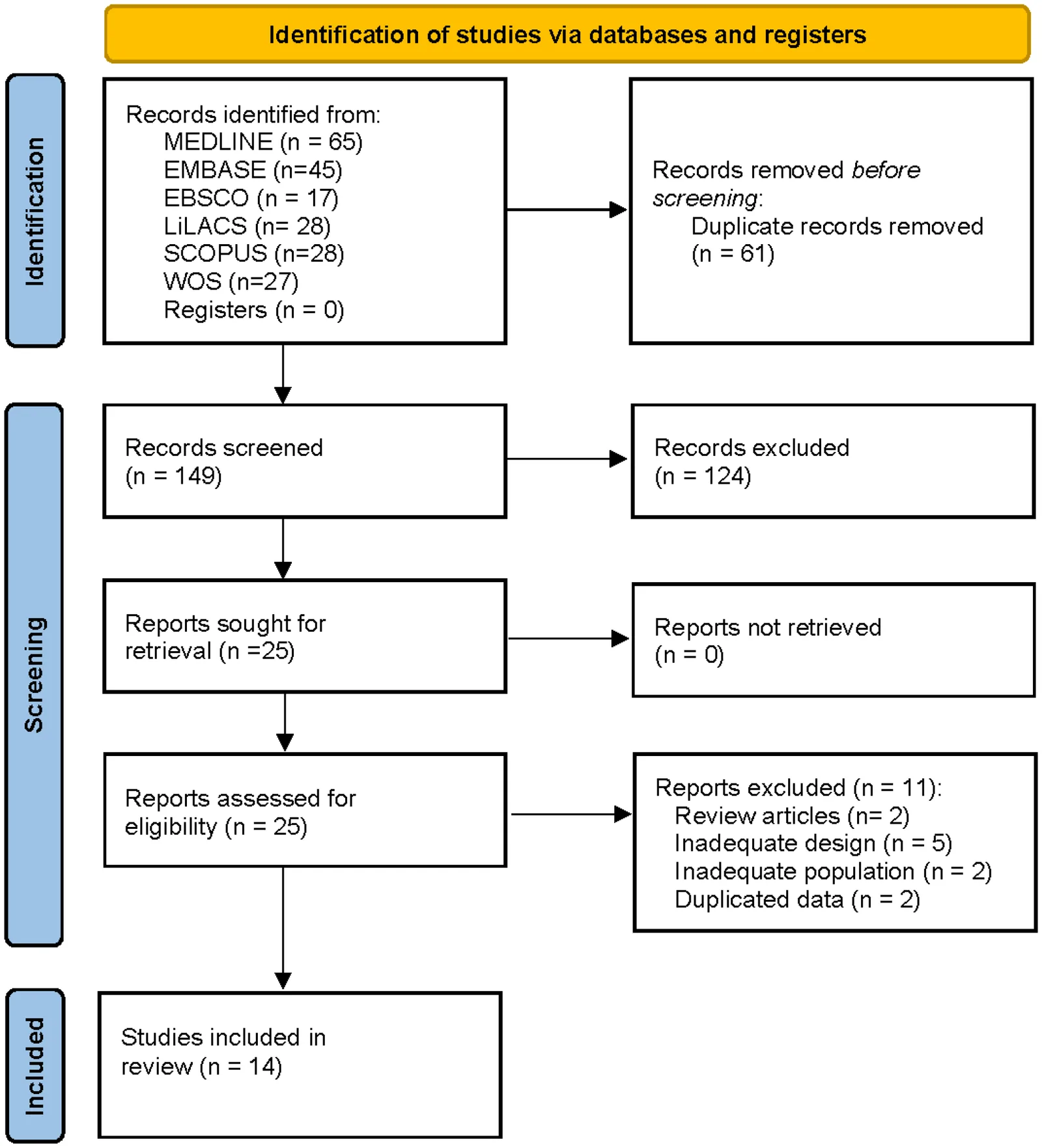
PRISMA flowchart.

Our bibliographic searches identified 14 studies that met all inclusion and exclusion criteria (**Figure 1**) and involved 1,323 participants (11–24). Of them, 10 studies reported the association between exposure to DAs and the occurrence of ICDs (11–18, 22, 24), whereas 6 reported the association between DAs and impulsiveness as measured by the Barratt Impulsiveness Scale (BIS-11) (16, 17, 19–21, 23) (**Table 1**). The 10 studies that reported on the association between DA exposure and ICDs contributed 13 independent comparisons to the meta-analysis, as three of them (13, 15, 16) included both patients with pituitary adenomas not exposed to DAs and healthy controls as control groups.

**Table 1:** Studies characteristics.

Study	Country	Design	Sample	ICD assessment tools	Rating scales	Summary of findings
Bancos 2013	USA	Cross- sectional	FPA+DAs: 63 FPA-DAs: 14 NFPA-DAs: 70	Modified SOGS, Modified HQ, Modified PQ	–	ICDs in 24.7% or 17.1% of patients exposed to DAs or not (p=0.31)
Beccuti 2020	Italy	Cross- sectional	FPA-DAs: 10 FPA+DAs: 111 NFPA-DAs: 48 NFPA+DAs: 21	QUIP-RS	–	ICDs in 52% vs 31% of patients exposed to DAs or not (p<0.01)
Hinojosa-Amaya 2020	Mexico	Cross- sectional	FPA-DAs: 9 FPA+DAs: 64 NFPA-DAs: 10 NFPA+DAs: 3 Other+DAs: 9 Other-DAs: 8	–	BIS	Higher scores were observed in DA-treated patients, but the difference was not significant
Sanjan 2023	India	Cohort (f-up=12 w)	FPA+DAs: 15 NFPA-DAs: 15	SOGS, MIDI, Modified PQ	BIS, IAS, K-SAS	No difference in the prevalence or ICDs or changes in the BIS
Celik 2018	Turkey	Cohort (f-up=52 w)	FPA+DAs: 25 NFPA-DAs: 31 HC: 32	MIDI, DSM-4/5	–	ICDs in 8% vs 0% in patients treated with DAs or not
De Sousa 2019	Australia	Cross-sectional	FPA+DAs: 107 NFPA+DAs: 6 HC: 99	QUIP-S, HBI	–	ICDs in 61.1% vs 42.4% of patients exposed to DAs or not (p<0.01)
Ozkaya 2020	Turkey	Cross-sectional	FPA+DAs: 40 NFPA-DAs: 38 NFPA+DAs: 38 HC: 32	MIDI, DSM-4/5	BIS	There were no differences between BIS in patients treated with DAs or not (p=0.72)
Ozdeniz 2023	Turkey	Cross-sectional	FPA-DAs: 20 FPA+DAS: 31 HC: 30	MIDI	BIS, ZSSS	ICDs affected 25.8%, 15%, or 16.7% of patients under DAs, or not, or HCs
Ke 2022	China	Cross-sectional	FPA-DAs: 12 FPA+DAs: 51 HC: 33	Mayo Clinic survey	–	ICDs affected 9.8%, 16.7%, or 9.1% of patients under DAs, or not, or HCs
Barake 2014	USA	Cross-sectional	FPA-DAs: 10 FPA+DAs: 10 NFPA-DAs: 10	ICDs Screening Questionnaire	BIS	There were no differences in the BIS score in patients exposed or not DAs, or HCs
Hamblin 2025	UK	Cross-sectional	FPA+DAs: 72 NFPA-DAs: 71	QUIP-S	–	ICDs in 46.5% vs 18.6% in patients treated with DAs or not (p<0.01)
Mohammad 2024	Iraq	Cross-sectional	FPA+DAs: 30 HC: 30	QUIP-S	–	ICDs in 30.0% vs 6.7% in patients treated with DAs or not (p<0.01)
Gokbulut 2026	Turkey	Cross-sectional	FPA+DAS: 34 HC: 35	–	BIS, IGT	No significant differences in BIS scores were observed
Zerbinati 2026	Italy	Cross-sectional	FPA+DAS: 63 HC: 63	PICS, QUIP-S	–	ICDs in 15.9% vs 6.3% in patients treated with DAs or in HCs (p<0.01)

FPA, Functioning Pituitary Adenoma; NFPA, Non-functioning Pituitary Adenoma; DAs, Dopamine agonists; HC, Healthy Controls; Other, Other pituitary disorders; f-up, follow-up period (only for cohort studies); SOGS, South Oaks Gambling Screening; QUIP-RS, Questionnaire for Impulsive-Compulsive Disorders in Parkinson’s Disease; MIDI, Minnesota Impulse Disorders Interview; HQ, Hypersexuality questionnaire; PQ, Punding Questionnaire; HBI, Hypersexual Behavior Inventory; IAS, Internet addiction score; ICD, Impulse-Control Disorders; IGT, Iowa Gambling Test; K-SAS, Kleptomania‐symptom assessment scale; PICS, Parkinson’s Impulse Control Scale; ZSSS, Zuckerman Sensation Seeking Scale.

Most studies were cross-sectional, except for two cohort studies (13, 21) (**Table 1**). Seven studies included only patients with prolactin-producing adenomas in the DA exposure group (11, 13, 15, 16, 18, 22, 24) and 7 used validated tools for the screening of ICDs (13, 14, 16–18, 22, 24) (**Table 1**). The most common DA was cabergoline, with weekly doses ranging from 0.38 to 17.50 mg and treatment durations of 12 to 416 weeks (**Table 2**). Differences in age and sex distribution were observed between participants exposed to dopamine agonists (DAs) and those not exposed (**Table 3**), reflecting variability in baseline characteristics across the included studies rather than risk associations. As shown in **Table 4**, all studies had a high risk of confounding and selection bias, except those by Beccuti et al. and Sanjan et al. The risk of outcome measurement was high in all cases because subjective patient-reported outcomes were used. In all cases, the risk of bias related to classification, deviations from intended interventions, missing data, or selective reporting was low.

**Table 2:** Exposure to dopamine agonists.

Study	Bromocriptine			Cabergoline			Quinagolide
	Cases	Mean dose (mg/w)	Mean exposure duration (weeks)	Cases	Mean dose (mg/w)	Mean exposure duration (weeks)	
Bancos 2013	22	26.25	94	41	0.98	94	–
Beccuti 2020	–	–	–	132	0.98	48	–
Hinojosa-Amaya 2020	6	52.50	UNK	70	1.96	UNK	–
Sanjan 2023	–	–	–	15	2.80	12	–
Celik 2018	–	–	–	25	3.64	12	–
De Sousa 2019	–	–	–	109	9.03	78	4
Ozkaya 2020	–	–	–	78	0.98	70	–
Ozdeniz 2023	3	8.75	UNK	27	5.46	UNK	1
Ke 2022	45	35.00	104	24	17.50	56	–
Barake 2014	1	UNK	UNK	9	1.12	33	–
Hamblin 2025	2	UNK	UNK	70	0.49	103	–
Mohammad 2024	–	–	–	30	4.20	151	–
Gokbulut 2026	–	–	–	34	0.38	36	–
Zerbinati 2026	–	–	–	63	0.75	416	–

**Table 3:** Study samples’ characteristics.

Study	Age			Male sex		
	DAs +	DAs -	P-value	DAs +	DAs -	P-value
Bancos 2013	54.6 ± 14.4	56.9 ± 14.2	0.33	47 (61%)	27 (40%)	<0.01
Beccuti 2020	48.9 (39.1-62.7)*	66.8 (52.0-73.1)*	<0.01	55 (42%)	33 (57%)	0.06
Hinojosa-Amaya 2020	42.79 ± 15.56	41.23 ± 15.09	0.65	34 (44.7%)	8 (29.6%)	0.17
Sanjan 2023	28.5 ± 1.9	42.2 ± 3.9	<0.01	6 (40%)	9 (57%)	0.573
Celik 2018	39.96 ± 10.11	49.5 ± 14.3	<0.01	7 (28%)	12 (39%)	0.23
De Sousa 2019	45.5 ± 16.5	41.2 ± 10.1	0.02	56 (49.6%)	46 (46.5%)	ns
Ozkaya 2020	40.4 ± 10.3	51.5 ± 13.8	<0.01	12 (30%)	16 (40%)	0.28
Ozdeniz 2023	36.8 ± 10.0	37.6 ± 12.1	0.977	3 (10%)	2 (10%)	0.88
Ke 2022	36.8 ± 11.7	42.7 ± 8.9	ns	12 (23%)	10 (80%)	ns
Barake 2014	38.8 ± 12.6	38.9 ± 12.2	ns	4 (40%)	2 (10%)	ns
Hamblin 2025	50 (21-77)*	60 (24-78)*	<0.01	38 (52.8%)	37 (52.1%)	0.99
Mohammad 2024	37.0 ± 11.8	33.2 ± 10.6	0.20	14 (46.7%)	18 (60.0)	0.10
Gokbulut 2026	42 (23-60)*	44 (18-59)*	0.50	11 (32.4%)	5 (42.8%)	0.26
Zerbinati 2026	48.7 ± 14.2	48.0 ± 15.7	0.91	26 (46.0%)	30 (47.6%)	0.87

DAs, Dopamine agonists; ns, not statistically significant. *median (percentiles 25-75).

**Table 4:** Risk of bias assessment.

Study	Confounding	Selection of participants	Classification of interventions	Deviations from intended interventions	Measure ment of outcomes	Missing data	Selection of the reported result
Bancos 2013	SERIOUS	SERIOUS	LOW	LOW	SERIOUS	LOW	LOW
Beccuti 2020	SERIOUS	SERIOUS	LOW	LOW	SERIOUS	LOW	LOW
Hinojosa-Amaya 2020	SERIOUS	SERIOUS	LOW	LOW	SERIOUS	LOW	LOW
Sanjan 2023	SERIOUS	LOW	LOW	LOW	SERIOUS	LOW	LOW
Celik 2018	SERIOUS	LOW	LOW	LOW	SERIOUS	LOW	LOW
De Sousa 2019	SERIOUS	SERIOUS	LOW	LOW	SERIOUS	LOW	LOW
Ozkaya 2020	SERIOUS	SERIOUS	LOW	LOW	SERIOUS	LOW	LOW
Ozdeniz 2023	SERIOUS	SERIOUS	LOW	LOW	SERIOUS	LOW	LOW
Ke 2022	SERIOUS	SERIOUS	LOW	LOW	SERIOUS	LOW	LOW
Barake 2014	SERIOUS	SERIOUS	LOW	LOW	SERIOUS	LOW	LOW
Hamblin 2025	SERIOUS	SERIOUS	LOW	LOW	SERIOUS	LOW	LOW
Mohammad 2024	SERIOUS	SERIOUS	LOW	LOW	SERIOUS	LOW	LOW
Gokbulut 2026	SERIOUS	SERIOUS	LOW	LOW	SERIOUS	LOW	LOW
Zerbinati 2026	SERIOUS	SERIOUS	LOW	LOW	SERIOUS	LOW	LOW

**Figure 2:**
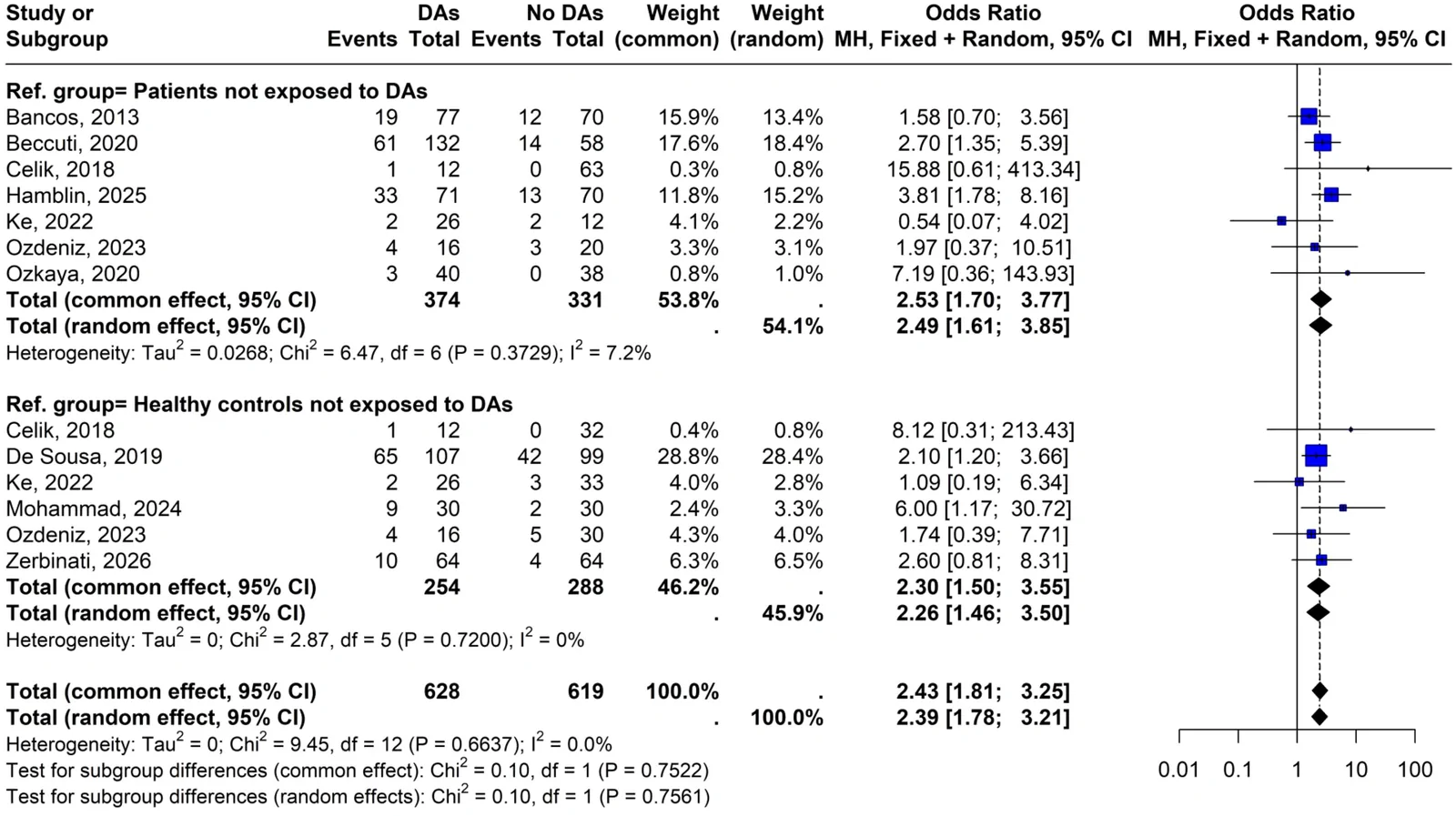
Forest plot of the frequency of impulse-control disorders in participants exposed to DAs or not. MH, Mantel-Haenszel analysis.

A random-effect analysis found a significant association between exposure to DAs and ICDs (OR, 95% CI: 2.39, 1.78-3.21; n=1247; p<0.01), **Figure 2**). Heterogeneity was not significant (I^2^ = 0%, p=0.58). There was no statistical difference in the effects of exposure to DAs compared to patients with pituitary adenomas not exposed to DAs or to healthy controls (Chi-sq=0.10, p=0.75). No evidence of a statistically significant small-study effect was found, but the limited number of available studies prevented a dependable evaluation of publication bias (Harbord test: t=1.08, df=11, p=0.30). The funnel plot is available as Online **Supplementary Material** (**Supplementary Figure E-2**).

The LOO analysis revealed that the global effect did not change significantly after excluding each study (**Supplementary Figure E-1**). When only studies that restricted the group exposed to DAs to patients with prolactin-producing adenomas were considered for the meta-analysis, the results remained essentially unchanged (2.41, 1.60-3.63; n=773; p<0.01; I^2^ = 0%; **Supplementary Figure E-5A**). Similarly, when only studies that employed validated tools for the screening of ICDs were considered, results were similar (2.75, 1.89-3.99; n=814; p<0.01; I^2^ = 0%; **Supplementary Figure E-5B**). Meta-regression analysis demonstrated that neither age (p=0.54), sex (p=0.39), cabergoline dose (p=0.07), nor treatment duration (p=0.74) modified the association between DAs exposure and the occurrence of ICDs (**Table 5**).

**Table 5:** Meta-regression results.

Variable	Beta (St.Error)	P-value
Mean age of the sample	0.17 (0.03)	0.54
Proportion of males in the sample	0.01 (0.01)	0.39
Mean Cabergoline dose in the sample	-0.52 (0.28)	0.07
Mean cabergoline treatment duration in the sample	0.0006 (0.0017)	0.74

When specific ICDs were analyzed, a significant association with DAs was found for hypersexuality (random-effects model, 3.11, 1.93-5.02, p<0.01, I^2^ = 0%) and punding (random-effects model, 2.53, 1.34-4.78, p<0.01, I^2^ = 27.6%), but not for pathological gambling (random-effects model, 1.94, 0.84-4.49, p=0.12, I^2^ = 0%) or compulsive shopping (random-effects model, 1.46, 0.59-3.36, p=0.41, I^2^ = 61.3%) (**Supplementary Figure E-3**, Online **Supplementary Material**).

Exposure to DAs and the BIS-11 score (mean difference, 95% CI = 1.42, -0.44 to 3.27, p=0.13, random-effects model) or its sub-scores were not associated (**Supplementary Figure E-4**, Online **Supplementary Material**).

## 4 Discussion

### 4.1 Summary of key findings

Our meta-analysis combined data from 1,323 participants across 14 observational studies worldwide. The odds of ICDs were approximately 2.4-fold higher in patients exposed to DAs for pituitary adenomas. Although individual studies were small and therefore had low statistical power, the overall evidence collected in this study suggests a consistent association (I² = 0%), which should be confirmed in larger, well-designed studies. There was a significant association between exposure to DAs and hypersexuality and punding, but not with gambling or compulsive shopping. These findings agree with large pharmacovigilance reports from other DAs-treated populations and reinforce the clinical relevance of ICDs beyond the Parkinson field (25).

Two systematic reviews have been previously conducted on this topic. In 2023, Hamblin and colleagues summarized the findings of 11 studies (26) narratively. They remarked that studies were largely retrospective, observational, and heterogeneous, and involved a limited number of patients treated with DAs. Results showed an ICD prevalence between 0-60% in patients with prolactinoma, and 5-23% in studies with at least five patients with acromegaly. The 2025 meta-analysis by Penchev and colleagues included 8 studies with 858 patients (27). They observed an increased frequency of ICDs in patients with pituitary adenomas exposed to DAs. Our meta-analysis included 14 studies with 1,323 patients, representing 54% more participants than the previous meta-analysis. We also compared patients with pituitary adenomas not exposed to DAs with healthy controls and analyzed the association between DAs and impulsivity. Finally, we used meta-regression to identify moderators. Our findings extend and update previous evidence.

### 4.2 Interpretation of the results

The development of these ICDs is related to dopamine’s role in the brain reward circuit (6). Dopamine modulates motivation, reinforcement learning, and pleasure-seeking behaviors in the mesolimbic pathway and nucleus accumbens, in particular (6). Overstimulation of D_2_ and D_3_ receptors may alter this balance, reducing inhibitory control over risky behaviors while simultaneously enhancing reward-seeking behaviors. This dysregulation appears to be a key mechanism underlying DA-associated ICDs. However, biological factors and innate variations in dopamine receptor function may influence individual susceptibility, so modifying the likelihood of developing ICDs as a side effect of treatment (6). The relationship between dopaminergic therapy and ICDs was first largely documented in patients with Parkinson’s Disease (25) and restless-leg syndrome (28), and is now being increasingly identified in patients with pituitary adenomas. ICDs affect up to 40% of individuals with PD and are closely linked to dopamine agonist use, younger disease onset, certain premorbid personality traits, and neuropsychiatric comorbidities (29). Neuropsychological studies show impairments in reflection impulsivity, temporal discounting, novelty seeking, risk evaluation, and inhibitory control, with mood disorders, sleep problems, apathy, and anxiety further increasing vulnerability. Despite growing awareness, ICDs continue to impose a substantial burden on patients and families and are associated with functional decline and reduced quality of life. The risk of ICDs in the Restless Legs Syndrome appears to be lower than for Parkinson’s disease, yet still significant and troublesome (28). Finally, it should be noted that ICDs also occur in a meaningful portion of the general population, with hypersexuality alone affecting an estimated 5–6% (30).

Most of the studies analyzed excluded patients with active psychiatric disorders, despite evidence linking a personal or family history of psychiatric illness with an increased vulnerability to DA-associated ICDs (31). A large Turkish cohort also identified male sex as a strong predictor of hypersexuality (2), which could not be confirmed in this study. Also, emerging genetic studies suggest that certain polymorphisms in dopaminergic and serotonergic genes may predispose individuals with prolactinomas to DA-related ICDs (32). Genetic susceptibility is further supported by twin studies showing a strong familial influence on compulsive gambling behavior, even in individuals not exposed to DAs (33). Altogether, these findings suggest a multifactorial model in which DAs’ interaction with pre-existing neurobiological vulnerability may contribute to the emergence of an ICD.

Our results showed no association between DAs use and impulsivity scores on the BIS-11. The often episodic, context-dependent, and reward-driven nature of DA-related behaviors may explain this, since the BIS-11 primarily measures stable personality traits (34). Some patients may find it hard to be utterly sincere when reporting their symptoms because of social, cultural, or age-related factors. Also, psychiatric history or substance use could affect impulsiveness, making results difficult to interpret. More specific tools, such as the QUIP questionnaire used in PD, are likely more useful for detecting these behaviors in clinical practice (20). The BIS-11 may be useful as a complementary, but not the only, screening method.

### 4.3 Limitations

The main limitation of our study stems from the comparison between treated and untreated patients. Since almost all patients with prolactin-producing pituitary adenomas eventually receive DAs, identifying patients among this group with no exposure to these drugs is difficult. Most of the studies analyzed were cross-sectional and had limited statistical power due to small sample sizes, making it difficult to examine specific subgroups by hormone levels, substance use, cultural background, and other factors. Indeed, only two of the 14 included studies employed a prospective cohort design, precluding inferences about incident risk. Also, the tools used to diagnose ICDs — QUIP, MIDI, SOGS — varied across studies, potentially leading to inconsistencies due to differences in detection characteristics. In any case, the results were consistent across studies, and the higher rate of ICDs in DAs-treated patients (compared with those with non-secreting tumors or healthy individuals) suggests an association with the medication. Future research should focus on larger prospective studies that use standard ICD definitions, include genetic information, and apply uniform criteria to measure DA exposure. Finally, we were unable to adequately analyze the relationships among DA type, treatment duration, and dosage due to low statistical power, underscoring the need for further research.

### 4.4 Implications and significance

From a clinical point of view, ICDs have a serious impact on patients’ lives, affecting their finances, relationships, and sometimes even leading to legal problems. Early detection of these symptoms is important, as they may improve with adjustments to the DA’s dose or by stopping treatment (2). We found no evidence that DAs dosage or duration modified the association with ICDs. However, some studies suggest that lowering the dose or stopping treatment may be beneficial (2). We encourage systematic screening of patients at the start of treatment and during follow-up visits, using tools such as the QUIP questionnaire. When clinically significant symptoms emerge or screening results are positive, referral for psychiatric or neuropsychiatric assessment is recommended to establish an appropriate diagnosis according to DSM-5 criteria (35). Cabergoline remains the first-line treatment for most patients because of its efficacy in reducing prolactin levels and tumor size. However, treatment decisions, including dose adjustments or alternative therapeutic strategies, should be individualized according to each patient’s risk–benefit profile, considering factors such as psychiatric history and potential susceptibility to behavioral adverse events (35).

## 5 Conclusion

Our findings suggest an association between exposure to DAs and ICDs, primarily accounted for by hypersexuality and punding, in patients with pituitary adenomas, which should be confirmed in larger, well-designed prospective studies. This said, it is essential to evaluate ICDs risk by considering psychiatric and family history, identifying individual risk factors, and securing multidisciplinary follow-up. This may help reduce potential harm and protect patients’ quality of life while maintaining the therapeutic benefits of dopaminergic treatment.

## Data Availability

The raw data supporting the conclusions of this article will be made available by the authors, without undue reservation.
