# Supplementary material for: A systematic review and meta-analysis on impulse-control disorders in patients with pituitary adenomas exposed to dopamine agonists
Source: Front Endocrinol (Lausanne). 2026 Aug 6;17:1869083. doi: 10.3389/fendo.2026.1869083 (PMC13491195; doi:10.3389/fendo.2026.1869083)
Supplement: Supplementary file 1 [file SupplementaryFile1.docx]

**Online Supplementary Material**

**Table E-1. Keyword search**

| **Language** | **Population** | **Intervention** | **Outcomes** |
| --- | --- | --- | --- |
| **Spanish** | Hiperprolactinoma  Hiperprolactinemia  Tumor/Adenoma hipofisario/pituitario | Agonistas dopaminérgicos  Agentes/tratamiento dopaminomimético/s | Trastorno del control de impulsos  Trastorno explosivo intermitente (TEI)  Juego patológico/Trastorno de juego/ludopatía  Piromanía  Tricotilomanía  Compras compulsivas  Hipersexualidad / trastorno hipersexual  Punding/conducta repetitiva |
| **English** | Hyperprolactinoma  Hyperprolactinemia  Pituitary/Hypophysis tumor adenoma | Dopamine agonists  Dopaminergic treatment | Impulse-control disorders  Intermittent explosive disorder (IED)  Pathological gambling/gaming disorder/gambling disorder  Pyromania  Trichotillomania  Compulsive shopping  Hypersexuality/hypersexual disorder  Punding/repetitive behavior |

**Search strings for each database**

**MEDLINE**

#1 hyperprolactin* OR ((pituitary* OR pituitary OR hypophys*) AND (tumor* OR adenoma* OR cancer* OR cancer))

#2 "dopamine receptor stimulating agent" OR "dopamine agonists" OR "dopaminergic treatment" OR dopamine

#3 "impulsive control disorder" OR "intermittent explosive" OR IED OR ((gambling OR gaming OR game) AND (patholog* OR disorder OR disease OR compulsiv* OR addic*)) OR pyromania OR trichotillomania OR ((shopping OR buying) AND (patholog* OR disorder OR disease OR compulsiv* OR addic*)) OR hypersexual* OR ((sex OR sexu*) AND (compulsiv* OR addic*))

#4 #1 AND #2 AND #3

**EMBASE**

#1 hyperprolactin* OR ((pituitary* OR pituitar* OR hypophys*) AND near AND (tumor* OR adenoma* OR cancer* OR cancer))

#2 "dopamine receptor stimulating agent" OR "dopamine agonists" OR "dopaminergic treatment"

#3 "Impulse control disorder" OR "intermittent explosive" OR IED OR (gambling OR gaming OR game) AND near AND (patholog* OR disorder OR disease OR compulsiv*) OR pyromania OR trichotillomania OR (shopping OR buying) AND near AND (patholog* OR disorder OR disease OR compulsiv*) OR hypersexual* OR (sexual* AND near AND (patholog* OR disorder OR disease OR compulsiv*)) OR punding OR (overeati* AND patholog* OR disorder OR disease OR compulsiv*)

#4 #1 AND #2 AND #3

**SCOPUS**

#1 TITLE-ABS-KEY ( hyperprolactin* OR ( ( pituit* OR hypophys* ) W/3 ( tumor OR cancer OR adenom* ) ) )

#2 TITLE-ABS-KEY ( dopamin* W/2 ( agonist* OR treatment ) )

#3 TITLE-ABS-KEY ( ( "impulse-control" W/2 disord* ) OR "intermittent explosive disorder" OR ied OR ( ( patholog* OR disorder* OR disease* ) W/2 ( gambl* OR gam* ) ) OR pyroman* OR trichotilloman* OR ( ( patholog* OR disorder* OR disease* ) W/2 ( shop* OR buy* ) ) OR ( ( patholog* OR disorder* OR disease* ) W/2 sexual* ) OR hypersexual* OR punding OR ( ( patholog* OR disorder* OR disease* ) W/2 repetitive ) OR "repetitive behaviour" )

#4 #1 AND #2 AND #3

**LiLACS**

#1 hiperprolactin* OR ((tumor OR cancer OR neoplasia) AND (pituit* OR hipofis*))

#2 ((agonista* OR tratamient*) AND dopamin*) OR dopaminomime*

#3 (control AND impulso*) OR "trastorno explosivo intermitente"OR IED OR ((problema* OR trastorno* OR desorden* OR patolog*) AND juego) OR ludopatia OR piroman* OR tricotilom* OR atracon* OR compra* OR hipersexual* OR ((problema* OR trastorno* OR desorden* OR patolog*) AND sex*) OR punding OR "conducta repetitiva"

#4 #1 OR (#2 AND #3)

**EBSCO**

#1 hyperprolactin* OR ( ( pituit* OR hypophys* ) N3 ( tumor OR cancer OR adenom* )

#2 dopamin* N3 ( agonist* OR treatment )

#3 ( "impulse-control" N3 disord* ) OR "intermittent explosive disorder" OR ied OR ( ( patholog* OR disorder* OR disease* ) N3 ( gambl* OR gam* ) ) OR pyroman* OR trichotilloman* OR ( ( patholog* OR disorder* OR disease* ) N3 ( shop* OR buy* ) ) OR ( ( patholog* OR disorder* OR disease* ) N3 sexual* ) OR hypersexual* OR punding OR ( ( patholog* OR disorder* OR disease* ) N3 repetitive ) OR "repetitive behaviour"

#4 #1 AND #2 AND #3

**WEB OF SCIENCE**

#1 hyperprolactin* OR ( ( pituit* OR hypophys* ) NEAR/3 ( tumor OR cancer OR adenom* )

#2 dopamin* NEAR/3 ( agonist* OR treatment )

#3 ( "impulse-control" NEAR/3 disord* ) OR "intermittent explosive disorder" OR ied OR ( ( patholog* OR disorder* OR disease* ) NEAR/3 ( gambl* OR gam* ) ) OR pyroman* OR trichotilloman* OR ( ( patholog* OR disorder* OR disease* ) NEAR/3 ( shop* OR buy* ) ) OR ( ( patholog* OR disorder* OR disease* ) NEAR/3 sexual* ) OR hypersexual* OR punding OR ( ( patholog* OR disorder* OR disease* ) NEAR/3 repetitive ) OR "repetitive behaviour"

#4 #1 AND #2 AND #3

**Figure E-1. Results of the Leave-One-Out sensitivity analysis.**

**Figure E-2. Funnel plot of the association between Impulse-Control Disorders and exposure to DAs**

**Figure E-3. Forest plot of the association between specific ICDs and exposure to DAs**

**PATHOLOGICAL GAMBLING**

**COMPULSIVE SHOPPING**

**HYPERSEXUALITY**

**PUNDING**

**Figure E-4. Forest plot of the association between exposure to DAs and Barratt impulsiveness scale total score and sub scores**

**TOTAL SCORE**

**TOTAL SCORE >=60**

**ATTENTION IMPULSIVENESS SCORE**

**MOTOR IMPULSIVENESS SCORE**

**NON-PLANNING IMPULSIVENESS SCORE**

**Figure E-5. Forest plot of the frequency of ICDs in subjects exposed to DAs or not. In panel A, only studies in which the exposed group comprised exclusively prolactin-producing prolactin adenomas were considered. In Panel B, only studies which used validated tools for the assessment of ICDs were considered.**

**A.**

**B.**
